# 5‐Hydroxy‐7‐methoxyflavone derivatives from *Kaempferia parviflora* induce skeletal muscle hypertrophy

**DOI:** 10.1002/fsn3.891

**Published:** 2018-11-20

**Authors:** Shintaro Ono, Naoki Yoshida, Daisuke Maekawa, Tomoya Kitakaze, Yasuyuki Kobayashi, Takehiro Kitano, Takanori Fujita, Hirotaka Okuwa‐Hayashi, Naoki Harada, Yoshihisa Nakano, Ryoichi Yamaji

**Affiliations:** ^1^ Division of Applied Life Sciences Graduate School of Life and Environmental Sciences Osaka Prefecture University Sakai Osaka Japan; ^2^ Japan Tablet Corporation Uji Kyoto Japan; ^3^ Center for Research and Development of Bioresources Osaka Prefecture University Sakai Osaka Japan

**Keywords:** 5‐hydroxy‐7‐methoxyflavone, Ca^2+^, *Kaempferia parviflora*, muscle hypertrophy, protein synthesis, senescence‐accelerated mouse

## Abstract

Skeletal muscle plays a critical role in locomotion and energy metabolism. Maintenance or enhancement of skeletal muscle mass contributes to the improvement of mobility and prevents the development of metabolic diseases. The extracts from *Kaempferia parviflora* rhizomes contain at least ten methoxyflavone derivatives that exhibit enhancing effects on ATP production and glucose uptake in skeletal muscle cells. In the present study, we investigated the effects of ten *K. parviflora*‐derived methoxyflavone derivatives (six 5,7‐dimethoxyflavone (DMF) derivatives and four 5‐hydroxy‐7‐methoxyflavone (HMF) derivatives) on skeletal muscle hypertrophy. Murine C2C12 myotubes and senescence‐accelerated mouse‐prone 1 (SAMP1) mice treated with methoxyflavones were used as experimental models to determine the effects of HMF derivatives on myotube diameter and size and muscle mass. The four HMF derivatives, but not the six DMF derivatives, increased myotube diameter. The 5‐hydroxyflavone, 7‐methoxyflavone, and 5,7‐dihydroxyflavone had no influence on myotube size, a result that differed from HMF. Dietary administration of the mixture composed of the four HMF derivatives resulted in increase in the soleus muscle size and mass in SAMP1 mice. HMF derivatives also promoted protein synthesis in myotubes, and treatment with the intracellular Ca^2+^ chelator BAPTA‐AM, which depletes intracellular Ca^2+^ levels, inhibited this promotion. Furthermore, BAPTA‐AM inhibited HMF‐promoted protein synthesis even when myotubes were incubated in Ca^2+^‐free medium. These results indicate that HMF derivatives induce myotube hypertrophy and that both the 5‐hydroxyl group and the 7‐methoxy group in the flavones are necessary for myotube hypertrophy. Furthermore, these results suggest that HMF‐induced protein synthesis requires intracellular Ca^2+^, but not extracellular Ca^2+^.

## 1 INTRODUCTION

Skeletal muscle is the most abundant tissue in healthy humans and contributes to not only locomotion, but also glycogen storage and metabolism of glucose and lipids. Skeletal muscle atrophy is caused by low habitual physical activity associated with a sedentary lifestyle or by immobilization due to prolonged bed rest, leading to loss of skeletal muscle mass (Bogdanis, 2012). Aging‐related skeletal muscle atrophy, called sarcopenia, also reduces physical activity, and ultimately results in immobilization (Kim & Choi, 2013; Narici & Maffulli, 2010). The reduction in muscle mass not only decreases physical activity but also increases the incidence of metabolic diseases such as obesity and type 2 diabetes (Koopman, Ly, & Ryall, 2014). Therefore, maintenance or enhancement of skeletal muscle mass would be a novel approach to restore decreased locomotion and energy metabolism. Recently, many studies have focused on the discovery and biochemical functions of beneficial food components that improve skeletal muscle health. However, more information regarding the relationship between structure and function of available food components and their impact on skeletal muscle is needed.

Prenatal growth of skeletal muscle involves the fusion of myoblasts into multinucleated myotubes that eventually form myofibers (Abmayr & Pavlath, 2012). The myotubes express myotube‐specific contractile proteins such as myosin heavy chain (MyHC) and tropomyosin (Devlin & Emerson, 1978). On the contrary, in adults, skeletal muscle mass is increased by the enlargement in the size of individual myofibers (called hypertrophy) (White, Bierinx, Gnocchi, & Zammit, 2010). A balance between muscle protein synthesis and degradation regulates skeletal muscle mass and increased protein synthesis causes muscle hypertrophy. Insulin‐like growth factor 1 increases protein synthesis, thereby promotes regeneration and hypertrophy of skeletal muscle (Gordon, Kelleher, & Kimball, 2013). Insulin‐like growth factor 1 induces Akt activation and subsequently activates the mechanistic target of rapamycin (mTOR) complex 1, which further causes hypertrophy and prevents atrophy (Bodine et al., 2001).

*Kaempferia parviflora* belongs to the Zingiberaceae family and has been used as a folk medicine and food in Thailand (Nakao et al., 2011). The extracts of *K. parviflora* rhizomes have various biological activities including: (a) inhibitory effects on P‐glycoprotein function (Patanasethanont et al., 2007); (b) anti‐plasmodial, anti‐fungal, and anti‐mycobacterial activities (Yenjai, Prasanphen, Daodee, Wongpanich, & Kittakoop, 2004); (c) cytotoxic effect on cancer cells (Yenjai & Wanich, 2010); (d) anti‐cholinesterase activity (Sawasdee, Sabphon, Sitthiwongwanit, & Kokpol, 2009); (e) anti‐allergic activity (Tewtrakul, Subhadhirasakul, & Kummee, 2008); (f) suppressive effect on the function of multidrug resistance‐associated protein; (g) anti‐gastric ulcer effect (Rujjanawate, Kanjanapothi, Amornlerdpison, & Pojanagaroon, 2005); (h) anti‐obesity effect (Akase et al., 2011); (i) aphrodisiac activity (Chaturapanich, Chaiyakul, Verawatnapakul, Yimlamai, & Pholpramool, 2012); and (j) enhancing effects on ATP production and glucose uptake in skeletal muscle cells (Toda et al., 2016). At least ten methoxyflavone derivatives (six 5,7‐dimethoxyflavone (DMF) derivatives and four 5‐hydroxy‐7‐methoxyflavone (HMF) derivatives) have been isolated from *K. parviflora* (Nakao et al., 2011). Recently, Lee, Kim, Kwon, Kim, and Hwang (2018) reported that *K. parviflora* extracts including 14.1% (w/w) DMF inhibit muscle atrophy in *ob/ob* mice. In this study, we report the effects of *K. parviflora*‐derived methoxyflavone derivatives, especially HMF derivatives, on hypertrophy of skeletal muscle cells and on skeletal muscle mass in senescence‐accelerated mouse‐prone 1 (SAMP1) mice. Furthermore, we analyzed the structure–activity relationship of the methoxyflavone derivatives on myotube hypertrophy and evaluated the effects of HMF derivatives on protein synthesis.

## 2 MATERIALS AND METHODS

### 2.1 Reagents

Mouse monoclonal anti‐MyHC (clone MF20 which recognizes all MyHC isoforms; clone BA‐D5 which recognizes MyHC type I) and anti‐tropomyosin (clone CH1) antibodies were obtained from Developmental Studies Hybridoma Bank, University of Iowa (Iowa City, IA). Horseradish peroxidase‐conjugated goat anti‐mouse IgG and anti‐rabbit IgG were from Bio‐Rad (Hercules, CA). Alexa Fluor 488‐conjugated goat anti‐mouse IgG was from Life Technologies (Gland Island, NY). 5‐Hydroxyflavone, 7‐methoxyflavone, and 5,7‐dihydroxyflavone were obtained from Tokyo Chemical Industry Co., Ltd. (Tokyo, Japan).

### 2.2 Preparation of methoxyflavones

**Figure 1:**
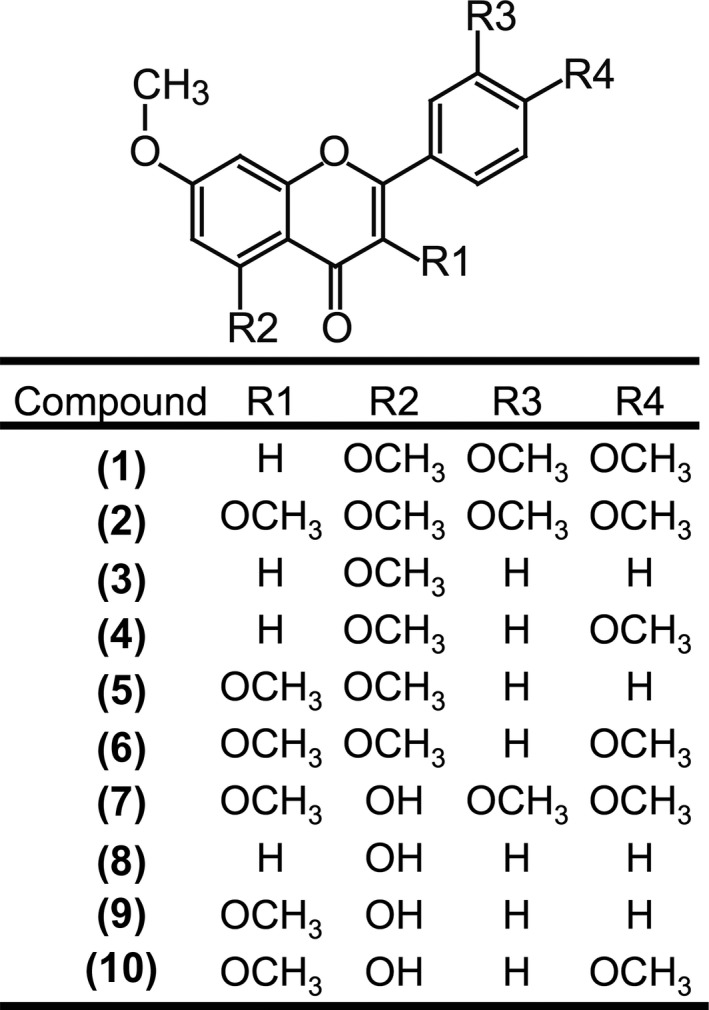
Chemical structures of methoxyflavones from *Kaempferia parviflora*. (**1**) 5,7,3′,4′‐tetramethoxyflavone, (**2**) 3,5,7,3′,4′‐pentamethoxyflavone, (**3**) 5,7‐dimethoxyflavone, (**4**) 5,7,4′‐trimethoxyflavone, (**5**) 3,5,7‐trimethoxyflavone, (**6**) 3,5,7,4′‐tetramethoxyflavone, (**7**) 5‐hydroxy‐3,7,3′4′‐tetramethoxyflavone, (**8**) 5‐hydroxy‐7‐methoxyflavone, (**9**) 5‐hydroxy‐3,7‐dimethoxyflavone, and (**10**) 5‐hydroxy‐3,7,4′‐trimethoxyflavone

The extracts from the rhizomes of *K. parviflora* were prepared, and ten methoxyflavone derivatives used in the study were purified according to methods described previously (Nakao et al., 2011). The chemical structures of the methoxyflavones are shown in Figure 1. For the animal experiments, the HMF derivative mixture including chemical compounds (**7**)–(**10**) was prepared. The dried slice rhizomes of *K. parviflora* were immersed in 70% (v/v) methanol for 1 hr by boiling. The filtrate was evaporated under reduced pressure at 50°C. The residue was poured into a glass column packed with silica gel 60 (Merck Millipore, Billerica, MA, USA) and equilibrated with n‐hexane. Separation was then performed with a mixture of n‐hexane and acetone (7:3). The HMF derivative mixture was obtained by evaporating the eluate. The HMF derivative mixture was composed of approximately 3.9% 5‐hydroxy‐3,7,3′,4′‐tetramethoxyflavone (**7**), 22.1% HMF (**8**), 20.6% 5‐hydroxy‐3,7‐dimethoxyflavone (**9**), and 29.7% 5‐hydroxy‐3,7,4′‐trimethoxyflavone (**10**).

### 2.3 Animals

The care of all animals was in accordance with the guidelines of the Animal Care and Use Committee of Osaka Prefecture University, which provided ethical approval for the present study (approval no. 26‐59). Male SAMP1 mice and their control senescence‐accelerated mouse‐resistant 1 (SAMR1) strains (16‐week‐old) were obtained from Japan SLC, Inc. (Shizuoka, Japan). Mice were individually housed in cages, and had free access to water and a non‐purified diet (CE‐2, Clea Japan, Inc., Tokyo, Japan) for 7 weeks. The mice were fed a purified diet (AIN‐76, where sucrose was replaced with cornstarch) for 1 week ad libitum. SAMP1 mice were randomly divided into two groups (*n* = 5 per group), one of which was fed the AIN‐76 experimental diet supplemented with 0.1% HMF derivative mixture for 4 weeks. The other group and the control SAMR1 mice were fed the AIN‐76 experimental diet without any supplementation for 4 weeks. These mice were kept under controlled temperature (23 ± 2°C), humidity (60 ± 10%), and lighting (12‐hr light–12 hr‐dark cycle).

### 2.4 Cell culture

Murine myoblast C2C12 cells were obtained from the RIKEN Cell Bank (Ibaraki, Japan). The cells were cultured in Dulbecco's modified Eagle's medium (DMEM) supplemented with 10% fetal bovine serum, 100 units/ml penicillin, and 100 μg/ml streptomycin (termed growth medium) at 37°C in a 5% CO_2_‐95% air atmosphere at 100% humidity as described previously (Ogawa et al., 2011). For differentiation, when myoblasts reached about 80%–90% confluency in the growth medium, they were shifted to DMEM supplemented with 2% horse serum and the above mentioned antibiotics (termed differentiation medium) for 8 days to differentiate them into myotubes. The differentiation medium was replaced at 48‐hr intervals.

### 2.5 Protein synthesis

C2C12 myotubes were cultured in serum‐free DMEM for 16 hr, followed by incubation in the presence of vehicle (DMSO), methoxyflavones (each 10 μM), or HMF derivative mix (5 μg/ml) for 1 hr. The myotubes were further incubated with puromycin at a final concentration of 1 μg/ml for 20 min. To determine the involvement of Ca^2+^ in HMF‐induced protein synthesis, myotubes were pre‐incubated with 5 μM BAPTA‐AM (Dojindo Laboratories, Kumamoto, Japan), an intracellular Ca^2+^ chelator, for 30 min, followed by incubation with HMF and puromycin labeling. Furthermore, to assess the involvement of extracellular Ca^2+^, myotubes were pre‐cultured in Ca^2+^‐free DMEM for 30 min, followed by incubation with HMF and puromycin labeling. The cells were then washed twice with phosphate‐buffered saline, lysed in RIPA buffer, and sonicated, as described previously (Kitakaze, Harada, Imagita, & Yamaji, 2015). Cell lysates were subjected to SDS‐PAGE and analyzed by western blot with anti‐puromycin and anti‐glyceraldehyde‐3‐phosphate dehydrogenase (GAPDH) antibodies (Yamaji et al., 2003). Immunoreactive proteins were incubated with horseradish peroxidase‐conjugated goat anti‐mouse IgG or anti‐rabbit antibodies, followed by reaction with Immobilon Western Chemiluminescent HRP Substrate (Millipore, Billerica, MA) and detection with LAS 4000 imaging system (GE Healthcare, Uppsala, Sweden).

### 2.6 Immunofluorescence analysis

The myotube size was determined as described previously (Ogawa et al., 2011). In brief, C2C12 myoblasts were cultured in differentiation medium for 8 days for differentiation into myotubes. The myotubes were cultured in the presence of DMSO (vehicle) or 10 μM flavones (e.g., methoxyflavones) for 4 days. Myotubes were fixed in 4% paraformaldehyde in phosphate‐buffered saline for 10 min, followed by permeabilization with 0.2% Triton X‐100 for 5 min. The cells were blocked with blocking solution, and incubated with anti‐MyHC antibody, followed by incubation with Alexa 488‐conjugated anti‐mouse IgG. The cells were stained with 4′,6‐diamidino‐2‐phenylindole (DAPI) in phosphate‐buffered saline. Fluorescent images were captured and analyzed using a BIOREVO BZ‐9000 fluorescence microscope (Keyence, Osaka, Japan). To determine the myotube size, the diameter of the short axes of myotubes was measured from five random fields. The mean myotube diameter was determined using more than 200 myotubes for each sample.

### 2.7 Western blot

For in vitro experiment, C2C12 myoblasts were cultured in differentiation medium for 8 days for differentiation into myotubes. The myotubes were cultured in differentiation medium in the presence of DMSO (vehicle) or 10 μM flavones (e.g., methoxyflavones) for 4 days. The cells were lysed in RIPA buffer (50 mM Tris‐HCl, pH 7.5, containing 150 mM NaCl, 1% sodium deoxycholate, 1% Triton X‐100, 0.1% SDS, 2 mM EDTA, 1 mM DTT, 1 mM phenylmethylsulfonyl fluoride, 1 μg/ml aprotinin, 10 μg/ml leupeptin, 1 mM sodium orthovanadate, 50 mM sodium fluoride, 10 mM sodium molybdate, and 10 mM sodium pyrophosphate) and sonicated. For in vivo experiment, the soleus muscle was homogenized in RIPA buffer, and the supernatant was collected following centrifugation. Cell lysates and tissue homogenates were subjected to SDS‐PAGE and analyzed by western blotting with mouse monoclonal anti‐MyHC (clone MF‐20 for in vitro experiment; clone BA‐D5 for in vivo experiment) (Developmental Studies Hybridoma Bank, University of Iowa, Iowa city, IA) antibodies and rabbit polyclonal anti‐GAPDH antibodies. The blots were then incubated with horseradish peroxidase‐conjugated goat anti‐mouse IgG and anti‐rabbit IgG, respectively. Immunoreactive proteins were reacted with Immobilon Western Chemiluminescent HRP substrate, followed by detection with an LAS4000 imaging system (GE Healthcare). The intensities of immunoreactive proteins were quantified by densitometry using ImageJ (version 1.43s, National Institute of Health).

### 2.8 Cross‐sectional area

The soleus muscles were dissected from male mice (*n* = 5 per hind limb), immediately fixed in 10% formalin, embedded in paraffin, sectioned at 6 μm, stained with hematoxylin and eosin, and examined under a light microscope (model BZ‐9000, Keyence, Osaka, Japan). The cross‐sectional area (CSA) (μm^2^) of muscle fibers was measured using Image J software (version 1.44p; National Institutes of Health, Bethesda, MD, USA). Mean fiber CSA was determined from approximately 250 fibers per soleus muscle. The distributions of the CSA of muscle fibers were plotted as frequency histograms.

### 2.9 Statistical analysis

One‐way analysis of variance (ANOVA) with the Dunnett's post hoc test was used in the experiments that had three or more groups. The significant difference between the two groups in animal experiments was determined using the Student's *t* test. Statistical analysis was performed using JMP statistical software version 8.0.1 (SAS Institute, Cary, NC, USA). Data are presented as mean ± *SD*. Differences were considered significant at *p *<* *0.05.

## 3 RESULTS

**Figure 2:**
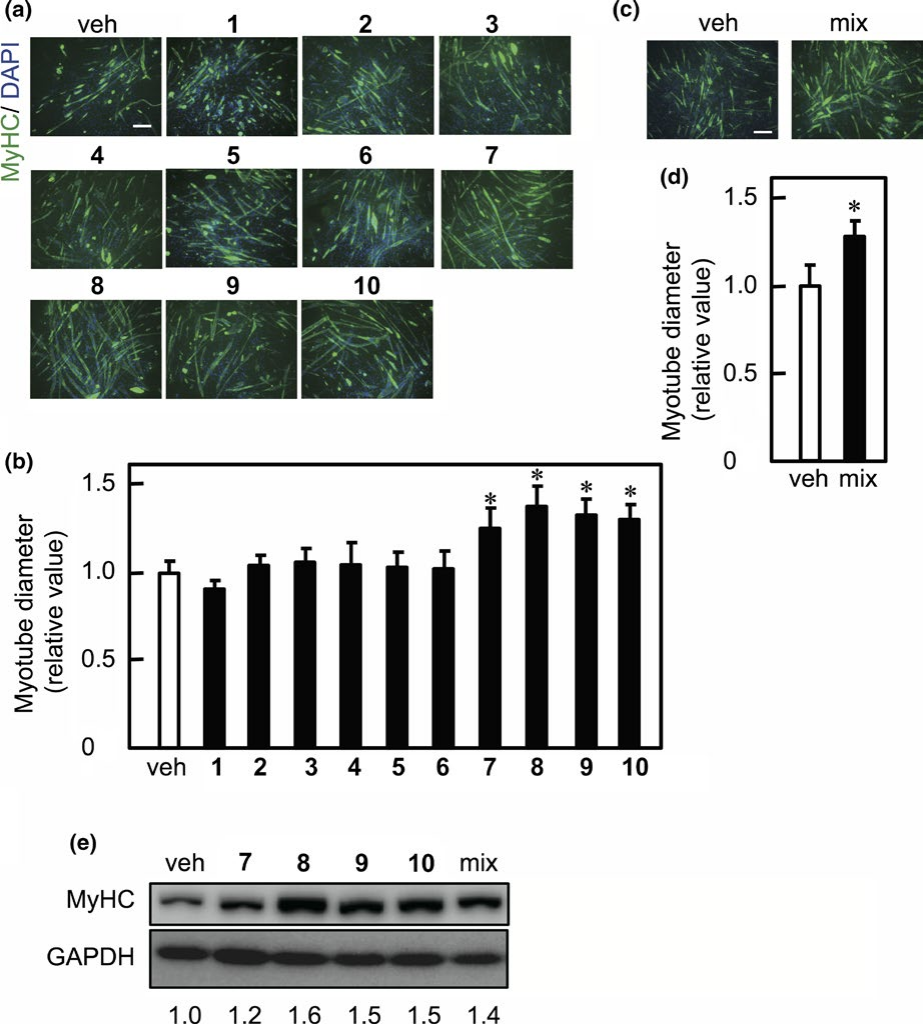
Effect of methoxyflavones on myotube diameter. (a) and (c) C2C12 myotubes were cultured in the presence of either vehicle (veh), methoxyflavone (**1**–**10**) (10 μM each), or HMF derivative mixture (mix) (5 μg/ml). Fixed myotubes were immunostained with anti‐MyHC antibody (clone MF‐20) and a fluorescently labeled secondary antibody (green). Nuclei were stained with DAPI (blue). Bars, 100 μm. (b) and (d) The diameter of short axes of myotubes was measured and graphically represented as mean ± *SD* (*n* = 3). The statistical significance when compared to the vehicle control was calculated using one‐way ANOVA with Dunnett's post hoc test. (*p <* *0.05). (e) C2C12 myotubes were cultured in the presence of either vehicle (veh), methoxyflavone (**1**–**10**) (10 μM each), or HMF derivative mixture (mix) (5 μg/ml). The cell lysates were analyzed by western blot using anti‐MyHC (MF‐20) and anti‐GAPDH antibodies. The band intensities of MyHC were normalized to those of GAPDH, with the relative values to vehicle indicated under the lower panel. (a)–(e) Each result is representative of two independent experiments

**Figure 3:**
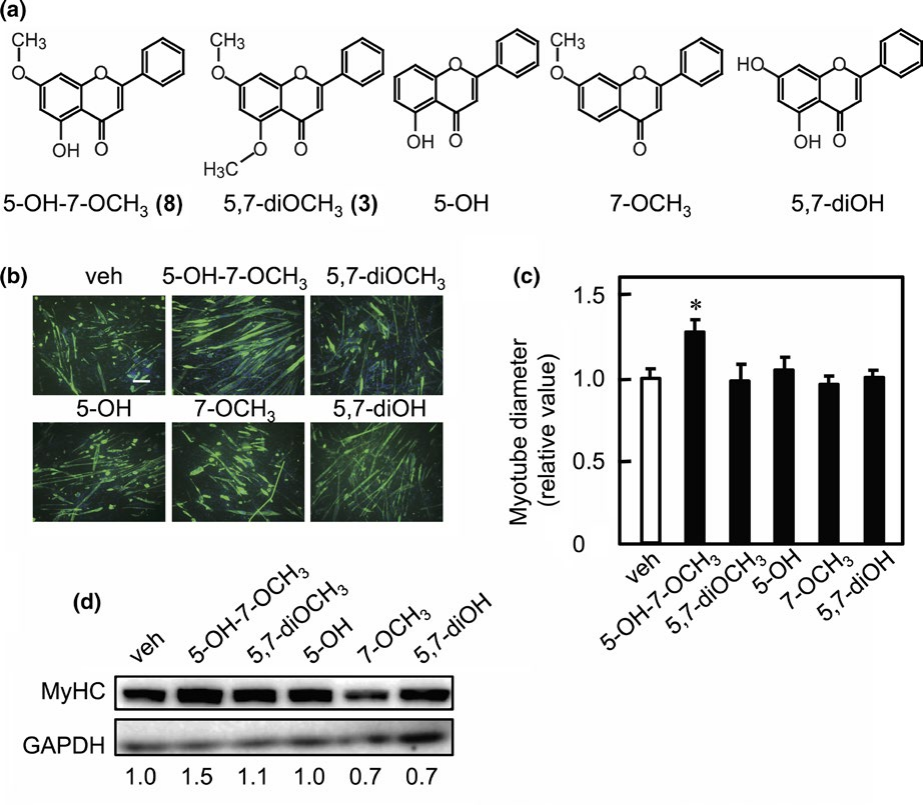
Structure–activity relationship of the 5‐hydroxyl and 7‐methoxy groups of HMF, and its effect on myotube diameter. (a) The chemical structure of 5‐hydroxy‐7‐methoxyflavone (**8**) (5‐OH‐7‐OCH_3_), 5,7‐dimethoxyflavone (**3**) (5,7‐diOCH_3_), 5‐hydroxyflavone (5‐OH), 7‐methoxyflavone (7‐OCH_3_), and 5,7‐dihydroxyflavone (5,7‐diOH). (b) C2C12 myotubes were cultured in the presence of either vehicle (veh) or the chemical compounds (10 μM each) shown in (a). Fixed myotubes were immunostained with anti‐MyHC antibody (MF‐20) and a fluorescently labeled secondary antibody (green). The nuclei were stained with DAPI (blue). Bars, 100 μm. (c) The myotube diameters were measured and graphically represented as mean ± *SD* (*n* = 3). Statistical significance when compared to the vehicle control was calculated using one‐way ANOVA with Dunnett's *post hoc* test. (*p <* *0.05). (d) C2C12 myotubes were cultured in the presence of either vehicle (veh) or the chemical compounds (10 μM each) shown in (a). Cell lysates were analyzed by western blot using anti‐MyHC (MF‐20) and anti‐GAPDH antibodies. The band intensities of MyHC were normalized to those of GAPDH. The relative values to vehicle are indicated under the lower panel. (b)–(d) Each result is representative of two independent experiments

To assess the effects of *K. parviflora*‐derived methoxyflavones on myotube hypertrophy, C2C12 myotubes were cultured in the presence of methoxyflavone derivatives. The myotubes were then labeled with anti‐MyHC antibody and were subjected to immunofluorescence analysis (Figure 2a). Individual HMF derivatives (**7**–**10**) increased the diameter of the short axis of myotubes, whereas individual DMF derivatives (**1**–**6**) had no influence on myotube size (Figure 2b). We also assessed the effects of HMF derivative mixture, which was composed of chemical compounds (**7**)–(**10**), on myotube diameter. We found that the HMF derivative mixture also increased myotube size (Figure 2c,d). To study the effects of the HMF derivatives and a mixture of these derivatives on MyHC expression, western blot analysis was performed using anti‐MyHC antibodies (clone MF‐20) that react with the total MyHC. Both the HMF derivatives and the mixture increased the expression level of MyHC (Figure 2e). Next, to determine the structure–activity relationship of the 5‐hydroxy and 7‐methoxy groups of the methoxyflavones, we used flavone compounds containing the 5‐hydroxy and 7‐methoxy groups individually or in combinations (Figure 3a) and determined their effect on myotube size. DMF (**3**), 5‐hydroxyflavone, 7‐methoxyflavone, and 5,7‐dihydroxyflavone had no influence on myotube size, while only HMF increased myotube size (Figure 3b,c). Furthermore, we assessed the relationship between the structure of HMF (**8**) and MyHC expression. We found that DMF (**3**), 5‐hydroxyflavone, 7‐methoxyflavone, and 5,7‐dihydroxyflavone had no influence on MyHC expression (Figure 3d). These results indicate that both the 5‐hydroxyl and 7‐methoxy groups in the derivatives are required to increase myotube size.

**Figure 4:**
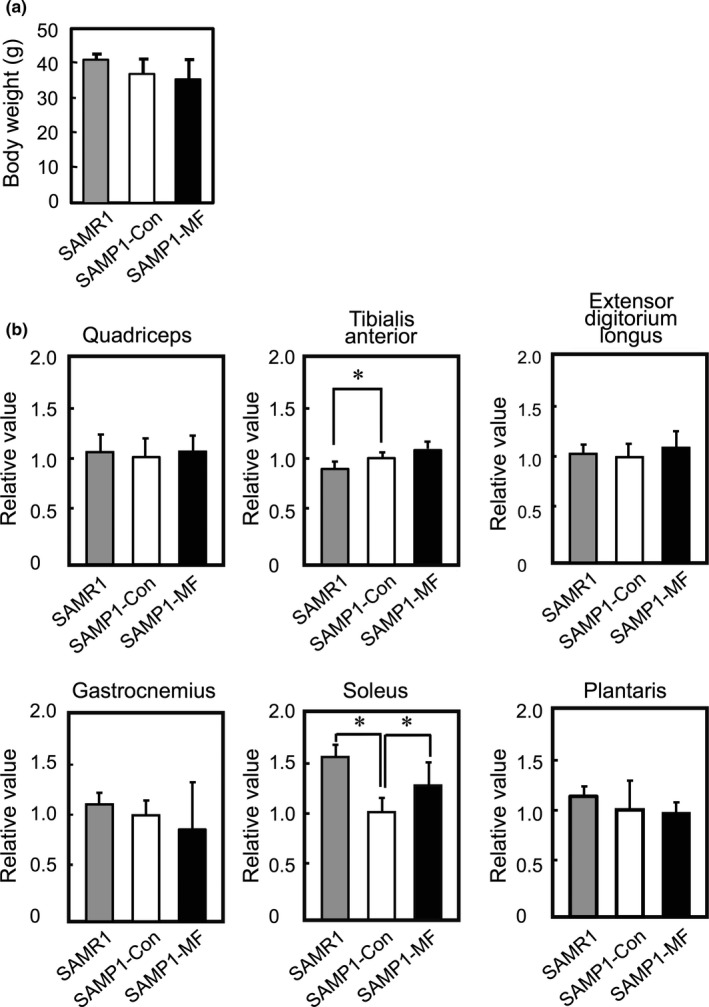
Effects of the HMF derivative mixture on skeletal muscle mass in SAMP1 mice. (a) SAMP1‐MF group (black bar) was administered HMF derivative mixture, while SAMR1 (gray bar) and SAMP1‐Con groups (white bar) were administered a control diet for 28 days. The graph represents the average body weight of mice in each group. (b) The ratio of skeletal muscle mass to body weight was determined for the different muscles. The statistical significance of the differences for the muscle mass/body weight ratio between SAMR1 and SAMP1‐Con and between SAMP1‐Con and SAMP1‐MF groups was analyzed using student's *t* test. The relative value to SAMP1‐Con was determined. The data are graphically represented as mean ± *SD* (*n* = 5 per group). (*p <* *0.05)

**Figure 5:**
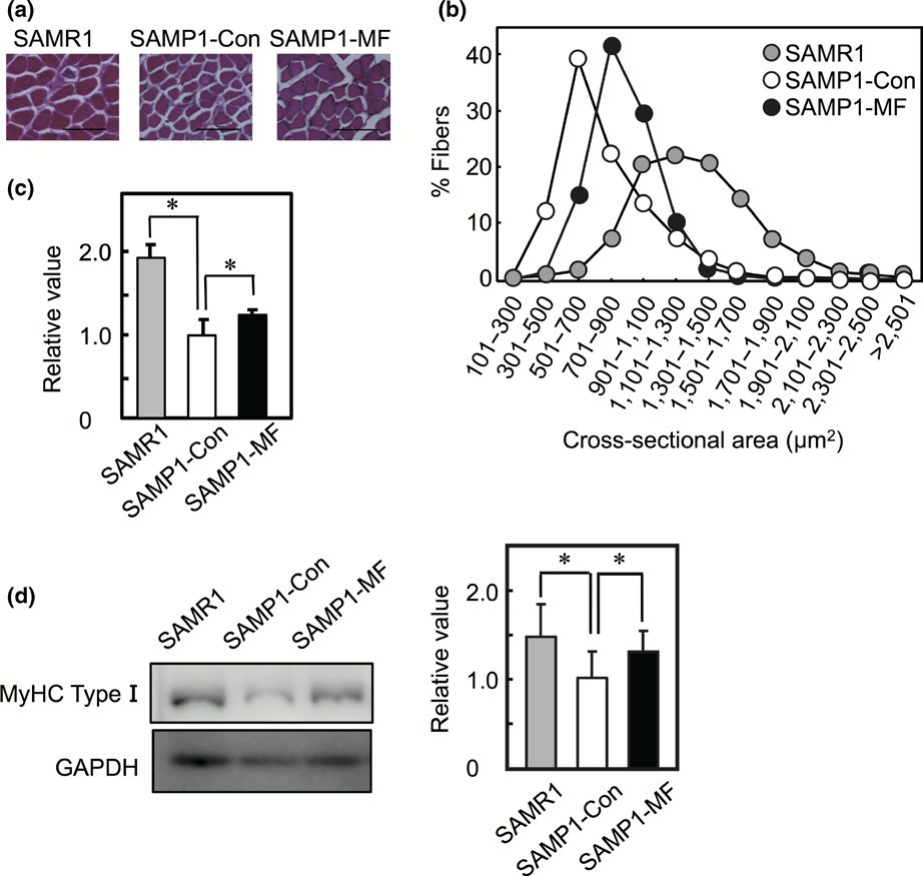
Cross‐sectional area of the soleus muscle when the HMF derivative mixture was administered to the SAMP1 mice. (a) Transverse section of the soleus muscle was stained with hematoxylin and eosin. Each image is representative of a soleus muscle from each group. Bars, 100 μm. (b) Distribution of CSA of the soleus muscles was examined (more than 1,000 fibers were measured in each group) in the SAMR1 (gray circle), SAMP1‐Con (white circle), and SAMP1‐MF (black circle) groups. (c) The average size of muscle fibers in SAMR1 and SAMP1‐MF groups was determined when compared to the SAMP1‐Con group. The data are represented as mean ± *SD* (*n* = 5 per group). (*p <* *0.05) (d) (left panel) Soleus muscles were homogenized, and tissue homogenates were analyzed by western blot using anti‐MyHC (BA‐D5) and anti‐GAPDH antibodies. (right panel) The band intensities of MyHC were normalized to those of GAPDH. The relative value to SAMP1‐Con was determined, and the data are represented as mean ± *SD* (*n* = 6 per group). (*p <* *0.05)

We assessed the effects of the HMF derivative mixture on muscle mass and myofiber size in vivo. SAMP1 mice were divided into two groups, one of which was fed the diet supplemented with the HMF derivative mixture, termed SAMP1‐MF. The other SAMP1 group (termed SAMP1‐Con) and the SAMR1 group were fed the control diet. Food consumption was not significantly different among SAMP1‐Con, SAMP1‐MF, and SAMR1 groups throughout the experiment. On day 28 after feeding, body weight was not significantly different among the three groups (Figure 4a). However, the ratio of muscle mass to body weight was significantly different between SAMR1 and SAMP1‐Con, and between SAMP1‐Con and SAMP1‐MF with respect to the soleus muscle, but not the quadriceps, tibialis anterior, extensor digitorium longus, gastrocnemius, and plantaris muscles (Figure 4b). The 28‐week‐old mice in the SAMP1‐Con group had an approximate reduction of 35% in the ratio of soleus muscle mass to body weight, when compared to those in the SAMR1 group. These results were consistent with the previous results (Murase, Haamizu, Ota, & Hase, 2008, 2009), which showed that the ratio of soleus muscle mass to body weight in SAMP1 is reduced by approximately 35% and 40% in 29‐week‐old and 31‐week‐old mice, respectively, when compared to mice in SMAR1 group. The CSA of each muscle fiber in the soleus muscle was stained with hematoxylin and eosin and quantitated (Figure 5a). The frequency distribution of CSA shifted toward larger sizes in SAMP1‐MF than in SAMP1‐Con (Figure 5b). The mean CSA of the soleus muscle was also higher in SAMP1‐MF group compared to the SAMP1‐Con group (Figure 5c). We also determined the expression level of slow MyHC (type I) as the soleus muscle is classified as a slow‐twitch muscle. The expression level of MyHC type I in soleus muscle was higher in SAMR1 and SAMP1‐MF groups than in SAMP1‐Con (Figure 5d). These results indicate that dietary administration of the HMF derivative mixture increases muscle mass and muscle fiber size and elevates the expression of MyHC type I in the soleus muscle.

**Figure 6:**
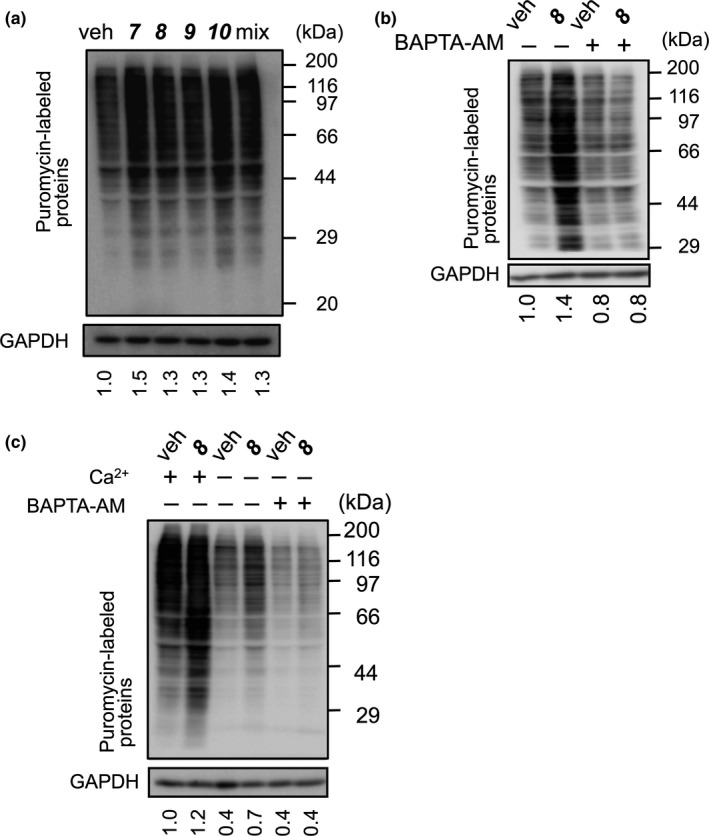
Involvement of Ca^2+^ in HMF‐induced protein synthesis. (a) C2C12 myotubes that were cultured in serum‐free DMEM were incubated with the HMF derivatives individually (compounds (**7**)–(**10**)) or with the HMF derivative mix (mix), followed by incubation with puromycin. (b) The myotubes, cultured as in (a), were pre‐incubated with BAPTA‐AM (5 μM), and subsequently incubated with HMF (**8**), followed by incubation with puromycin. (c) The myotubes were cultured in Ca^2+^‐free and serum‐free DMEM in the presence or absence of BAPTA‐AM, and incubated with HMF (**8**), followed by a subsequent incubation with puromycin. (a), (b), and (c) Cell lysates were prepared, and then analyzed by western blot with anti‐puromycin and anti‐GAPDH antibodies. Anti‐GAPDH antibody was used as the loading control. (a)–(c) The band intensities of puromycin were normalized to those of GAPDH, and the relative values to vehicle were indicated under the lower panel. Each result is representative of two independent experiments

We then determined the effects of HMF derivatives (**7**–**10**) on protein synthesis in myotubes by puromycin incorporation as a marker. The four HMF derivatives, both individually and as a mixture, promoted protein synthesis in myotubes (Figure 6a). To examine the role of intracellular Ca^2+^, the myotubes were incubated with HMF (**8**) in the presence or absence of the intracellular Ca^2+^ chelator BAPTA‐AM. We observed that HMF‐induced protein synthesis was inhibited in the presence of BAPTA‐AM (Figure 6b). To assess the effect of extracellular Ca^2+^ on HMF‐induced protein synthesis, myotubes were incubated with HMF in a Ca^2+^‐free medium. HMF promoted protein synthesis even in the absence of extracellular Ca^2+^ and addition of BAPTA‐AM inhibited its promotion (Figure 6c). These results suggest that HMF‐promoted protein synthesis requires intracellular Ca^2+^, and not extracellular Ca^2+^.

## 4 DISCUSSION

*Kaempferia parviflora* has at least ten methoxyflavone derivatives. The ethanolic extract of *K. parviflora* and some of its methoxyflavone derivatives have various biological activities including enhancement of energy metabolism in C2C12 myoblasts and myotubes (Toda et al., 2016). Recently, Lee et al. (2018) reported that the ethanolic extract of *K. parviflora* inhibits muscle atrophy in *ob/ob* mice. However, the structure–activity relationship of the methoxyflavone derivatives on myotube hypertrophy was still unclear. Skeletal muscle exhibits plasticity, wherein resistant training causes muscle hypertrophy while physical inactivity causes muscle atrophy. The size of myotubes determines skeletal muscle mass (Rai, Nongthomba, & Grounds, 2014). Loss of skeletal muscle mass leads to the most recognizable manifestations, such as muscle weakness and mobility impairments. In the present study, we determined the effects of methoxyflavones on myotube size.

Data obtained in this study showed that the HMF derivatives (**7**–**10**), but not the DMF derivatives (**1**–**6**), increased myotube size in C2C12 cells. In addition, the dietary HMF derivative mixture increased soleus muscle mass in SAMP1 mice in vivo. In this study, we have addressed the importance of the moieties in the methoxyflavone structure that influences myotube hypertrophy. Data regarding the structure–activity relationships demonstrated that both the hydroxy group at the 5‐position and the methoxy group at the 7‐position of the methoxyflavones contributed to myotube hypertrophy. These results suggest that HMF derivatives function as hypertrophy inducers through a common target protein. However, compounds (**8**), (**10**), and (**3**), but not compounds (**7**), (**9**), (**2**), (**4**), and (**6**), induce glucose transporter 4 and peroxisome proliferator‐activated receptor γ coactivator‐1α expression in myoblasts, indicating that there is no structure–activity relationship in mediating this function (Toda et al., 2016). Taken together, the target proteins with which HMF interacts are likely to be different for induction of hypertrophy and for increasing gene expression.

The 5,7‐dihydroxyflavone (chrysin) at a 10 μM concentration had no influence on myotube size when myotubes were cultured in its presence for 4 days (Figure 3c). In contrast, 5,7,4′‐trihydroxyflavone (apigenin) increased myotube size when C2C12 myoblasts differentiated into myotubes in its presence (2.5 or 5 μM) for 6 days (Jang et al., 2017). Furthermore, apigenin increased the phosphorylation of Akt and p70S6K, which is the substrate of mTOR complex 1, when myoblasts are differentiated in its presence for 2 days. Dietary apigenin activated mTOR signal and induced hypertrophy in the quadriceps muscle. Taken together, the hydroxy group at the 4′‐position of apigenin may play a critical role in muscle hypertrophy. On the other hand, dietary *K. parviflora* extracts including 14.1% (w/w) DMF increases skeletal muscle mass in *ob/ob* mice and activates mTOR signaling. However, it remains unclear whether DMF directly acts on mTOR signaling in skeletal muscle (Lee et al., 2018). In our study, HMF did not induce the phosphorylation of Akt and p70S6K until 60 min after myotubes were stimulated with it (data not shown). Presently, however, we cannot deny that there is no involvement of the Akt‐mTOR signal in HMF‐promoted protein synthesis. We are also unable to define how HMF promotes protein synthesis. We are therefore attempting to assess the effect of HMF on mTOR signaling over longer time periods, following stimulation, as well as its effect on several other signaling pathways.

Treatment with BAPTA‐AM inhibited the HMF‐promoted protein synthesis even in the absence of Ca^2+^ in the extracellular space. This suggests the involvement of intracellular Ca^2+^, but not extracellular Ca^2+^, in mediating the effect exhibited. Skeletal muscle mass is regulated by the balance between synthesis and degradation of muscle proteins, and increased protein synthesis leads to muscle hypertrophy. Ca^2+^ plays a role in highly multi‐functional intracellular signaling pathways (Berridge, Bootman, & Roderick, 2003). Several studies have reported that Ca^2+^ signaling is involved in muscle hypertrophy. Mechanical overload on muscle activates transient receptor potential cation channel vanilloid 1, a non‐selective cation channel. This subsequently increases intracellular Ca^2+^ levels, leading to muscle hypertrophy through activating mTOR (Ito, Ruegg, Kudo, Miyagoe‐Suzuki, & Takeda, 2013). HMF derivatives may therefore regulate muscle hypertrophy through Ca^2+^ signaling.

The present results demonstrate that HMF derivatives of *K. parviflora*, but not the DMF derivatives, promote myotube hypertrophy and increase myofiber size in the soleus muscle of SAMP1 mice, when they are 28 weeks old. This age corresponds to middle‐aged mice rather than aged mice. It would therefore be interesting to determine whether the MF derivative mixture exhibits any beneficial effects on skeletal muscle in aged mice. Myotube hypertrophy is also induced by resistance exercise. Recently, it was reported that consuming *K. parviflora* extract improved the physical fitness of a soccer player (Promthep, Eungpinichpong, Sripanidkulchai, & Chatchawan, 2015) and the elderly (Wattanathorn et al., 2012). Further studies should therefore address how *K. parviflora* extract, especially HMF derivatives, may positively impact skeletal muscle health. Overall, our results suggest that these derivatives may serve as a promising dietary supplement to maintain and enhance skeletal muscle mass, via inducing hypertrophy.

## 5 CONCLUSION

Our results indicated that the HMF derivatives, but not the DMF derivatives, promote myotube hypertrophy in C2C12 cells and increased myofiber size in the soleus muscle of SAMP1 mice. Data regarding the structure–activity relationships demonstrated that both the hydroxy group at the 5‐position and the methoxy group at the 7‐position of methoxyflavones play a critical role in myotube hypertrophy. Furthermore, HMF derivatives were observed to promote protein synthesis in myotubes through Ca^2+^ signaling.

## CONFLICT OF INTEREST

Osaka Prefecture University and Japan Tablet Corporation hold a patent on the effect of methoxyflavones on skeletal muscle hypertrophy. HO‐H, TF, and RY are the inventors of the patent. TF is an employee of Japan Tablet Corporation. HO‐H was an employee of Japan Tablet Corporation. RY received scholarship donation from Japan Tablet Corporation.

## AUTHOR CONTRIBUTION

RY designed the study. SO, NY, DM, TK, YK, and TK conducted the experiments. TF and HO‐H purified methoxyflavone derivatives from *K. parviflora*. RY, NH, and YN analyzed the data and performed statistical analysis. RY wrote the manuscript.

## ETHICAL STATEMENTS

This study was approved by the Institutional Review Board of Osaka Prefecture University.
